# Resveratrol suppresses glial activation and alleviates trigeminal neuralgia via activation of AMPK

**DOI:** 10.1186/s12974-016-0550-6

**Published:** 2016-04-19

**Authors:** Yan-jing Yang, Liang Hu, Ye-peng Xia, Chun-yi Jiang, Chen Miao, Chun-qing Yang, Miao Yuan, Lin Wang

**Affiliations:** Jiangsu Key Laboratory of Oral Disease, Nanjing Medical University, 140 Hanzhong Road, Nanjing, Jiangsu 210029 People’s Republic of China; Jiangsu Key Laboratory of Neurodegeneration, Department of Pharmacology, Nanjing Medical University, 140 Hanzhong Road, Nanjing, Jiangsu 210029 People’s Republic of China; Department of Pathology, First Affiliated Hospital of Nanjing Medical University, 300 Guangzhou Road, Nanjing, Jiangsu 210029 People’s Republic of China

**Keywords:** TN, Resveratrol, AMPK, Glia activation, Cytokines, MAPK

## Abstract

**Background:**

Glial activation and neuroinflammation in the spinal trigeminal nucleus (STN) play a pivotal role in the genesis and maintenance of trigeminal neuralgia (TN). Resveratrol, a natural compound from grape and red wine, has a potential anti-inflammatory effect. We hypothesized that resveratrol could significantly suppress neuroinflammation in the STN mediated by glial activation and further relieve TN. In this study, we evaluated whether resveratrol could alleviate trigeminal allodynia and explore the mechanism underlying the antinociceptive effect of resveratrol.

**Methods:**

Animals were orally injected with resveratrol after chronic constriction injury (CCI) of the infraorbital nerve. Mechanical thresholds of the affected whisker pad were measured to assess nociceptive behaviors. The STN was harvested to quantify the changing levels of p-NR1, p-PKC, TNF-α, and IL1-β by western blotting and detect the expression of calcitonin gene-related peptide (CGRP) and c-Fos by immunofluorescence. Glial activation was observed by immunofluorescence and western blotting. Mitogen-activated protein kinase (MAPK) phosphorylation in vivo and in vitro was examined by western blotting.

**Results:**

We found that resveratrol significantly attenuated trigeminal allodynia dose-dependently and decreased the increased expression of CGRP and c-Fos in the STN. Additionally, resveratrol showed an inhibitory effect on CCI-evoked astrocyte and microglia activation and reduced production of pro-inflammatory cytokines in the STN. Furthermore, the antinociceptive effect of resveratrol was partially mediated by reduced phosphorylation of MAP kinases via adenosine monophosphate-activated protein kinase (AMPK) activation.

**Conclusions:**

AMPK activation in the STN glia via resveratrol has utility in the treatment of CCI-induced neuroinflammation and further implicates AMPK as a novel target for the attenuation of trigeminal neuralgia.

## Background

Trigeminal neuralgia (TN) is the most common type of neuropathic pain which is distributed at the branches of the trigeminal nerve. It is characterized by unilateral pain attacks which are sharp, shooting, lancinating, electric shock-like, burning, and excruciating. Therefore, TN is known as one of the most painful complaints of humans. However, the underlying mechanisms of TN have not been completely elucidated, and the outcomes of pharmacological or surgical treatments are often disappointing [1]. Thus, it is urgent to develop new approaches and discover new agents to treat TN.

Previous studies related to the therapy of neuropathic pain focus on the primary sensory neurons and their influence on the activity of spinal dorsal horn neurons [2, 3]. However, emerging evidence implicates that both microglia and astrocytes which are robustly activated and persist in the spinal trigeminal nucleus play an important role in TN [4, 5]. Activation of glial cells in the central nervous system (CNS) continues to release pro-inflammatory cytokines, such as interleukin-1β (IL-1β), interleukin-6 (IL-6), and tumor necrosis factor-α (TNF-α), which increase pain hypersensitivity [6–9] and participate in the pathogenesis of neuropathic pain [10]. These upregulated cytokines construct a cytokine network and elicit chronic neuroinflammation leading to neuropathic pain [11, 12]. Therefore, targeting inhibition of glial activation is a potentially novel treatment target for TN.

Resveratrol is a natural plant antibiotic found in various plants and fruits, especially abundant in grapes and red wine [13, 14]. Not only is resveratrol widely known for its anti-oxidant and anti-inflammation properties, but it also presents the neuroprotective benefits according to ameliorating kainate-induced excitotoxicity [15] and improves pathological and behavioral outcomes in various types of CNS injuries including stroke [16], traumatic brain injury [17], and spinal cord injury [18]. The mechanism of resveratrol-induced neuroprotection is not clear, but many of its benefits are thought to activate adenosine monophosphate (AMP)-activated protein kinase (AMPK) [19]. Multiple lines of evidence have indicated that AMPK is emerging as a vital target to promote chronic pain through the sensitization of peripheral nociceptors [20]. However, few relevant reports assess the inhibition of resveratrol on glial activation via upregulating AMPK to alleviate mechanical allodynia in TN.

Herein, we tested the hypothesis that AMPK could represent a novel and efficacious opportunity for the treatment of TN. In the present study, we firstly determined whether oral administration of resveratrol, as an AMPK activator, could relieve mechanical allodynia of the trigeminal nerve. Further, we investigated the effect of resveratrol on glial activation and consequently examined its underlying mechanism. The results showed that resveratrol could significantly attenuate trigeminal neuralgia behaviors in the chronic constriction injury (CCI) model. Moreover, activated microglia and astrocytes markedly increased in the CCI model, whereas resveratrol decreased the activation of microglia and astrocytes and decreased the release of inflammatory cytokines in the CCI rat. Finally, we found that mitogen-activated protein kinase (MAPK) signal pathway was involved in the analgesic effect of resveratrol, and the involvement of MAPK signal pathway was confirmed in BV-2 cell line and primary astrocytes. Based on these findings, our postulation was that AMPK plays an analgesic action in TN by inhibiting the activation of microglia and astrocytes.

## Methods

### Ethics statement

All procedures were strictly performed in accordance with the regulations of the ethics committee of the International Association for the Study of Pain and the Guide for the Care and Use of Laboratory Animals (The Ministry of Science and Technology of China, 2006). All animal experiments were approved by Nanjing Medical University Animal Care and Use Committee and were designed to minimize suffering and the number of animals used.

### Animals

Sprague-Dawley male rats (200–220 g at the start of the experiment) were provided by the Experimental Animal Center at Nanjing Medical University, Nanjing, China. Five to six animals were housed per cage under pathogen-free conditions with soft bedding under controlled temperature (22 ± 2 °C) and photoperiods (12:12-h light-dark cycle). They were allowed to acclimate to these conditions for at least 2 days before inclusion in experiments. Animals were randomly divided into groups (*n* = 8). The sample size was designed based on the previous experience and to be limited to the minimal as scientifically justified. For each group of experiments, the animals were matched by age and body weight. All surgeries were performed under anesthesia with pentobarbital (Sigma, USA, 50 mg/kg, intraperitoneally (i.p.)).

### Drugs and reagents

Fetal bovine serum (FBS) and other cell culture media and supplements were purchased from Hyclone (USA). Compound C and resveratrol were purchased from Sigma. Primary antibody p-NR1 (Ser897) was purchased from Millipore; TNF-α, p-ERK1/2 (Thr202/Tyr204), p-JNK (Thr183/Tyr185), p-p38 (Thr180/Tyr182), and p-PKCγ (Thr514) were purchased from Cell Signaling Technology; glyceraldehyde-3-phosphate dehydrogenase (GAPDH) was purchased from Sigma; glial fibrillary acidic protein (GFAP) and IL-1β were purchased from Santa Cruz Biotechnology; and IBA-1 was purchased from Abcam. Secondary antibodies were purchased from Cell Signaling Technology. All other chemicals were purchased from Sigma Chemical Co.

### Surgery

Rats were anesthetized with sodium pentobarbital (50 mg/kg, i.p.), and all surgeries were conducted in sterile conditions under a surgical microscope. The hair on the top of the head was shaved, and the rat was placed in a stereotaxic frame. Ophthalmic cream was applied to the corner of both eyes to prevent drying damage. An anterior-posterior 15-mm skin incision was made at midline of the head. The infraorbital muscle was gently dissected from the bone until the orbit could be gently retracted. A piece of gelfoam or a tiny cotton ball was packed into the orbital cavity to minimize bleeding. The infraorbital nerve can be seen deep within the orbital cavity, lying in the infraorbital bony fissure. The infraorbital nerve was dissected free from the bone at its most rostral extent in the orbital cavity, and a single 2-mm length of chromic gut suture (4-0) was inserted between the infraorbital nerve and the maxillary bone. In the sham operation control group, only skin incision and muscle dissection were performed. The nerve was not touched, and no chromic gut suture was inserted. All skin incisions were sutured with 4-0 nylon non-absorbable monofilament.

### Assessment of mechanical allodynia on the whisker pad

Mechanical sensitivity of the whisker pad, the infraorbital nerve receptive field, was measured with a series of von Frey fiber filament (Stoelting, Wood Dale, IL) by modified up-down method. Rats were handled several times before experiments. One experimenter held the rat with two hands in insulating cotton gloves until the animal was calm. The animal moved freely in the holder’s hands with its head exposed. During testing, one experimenter slightly restrained the rat in their hands so that another experimenter could accurately apply the von Frey filament onto the center of the rat whisker pad, both ipsilateral and contralateral to the surgery site. For consistency of results, each filament was applied five times at intervals of a few seconds. If head withdrawal was observed at least three times after probing with a filament, the rat was considered responsive to that filament according to the up-down method. For this approach, whenever a positive response to the mechanical stimulus occurred, the next weaker von Frey filament was applied. If no positive response was evoked, the next stronger filament was applied. Testing proceeded in this manner until four fibers were applied after the first one successfully caused positive responses. This allowed estimation of the 50 % mechanical withdrawal threshold (in gram) using a curve-fitting algorithm.

### Cell cultures

Microglial BV-2 cells were incubated under humidified 5 % CO_2_ and 95 % O_2_ at 37 °C in Dulbecco’s modified Eagle’s medium (DMEM, Invitrogen, USA) containing 10 % FBS and 1 % streptomycin and penicillin (Invitrogen). Twenty-four hours before experimentation, the culture medium was replaced by 0.5 % FBS high-glucose DMEM. Then, the cells were stimulated with IL-1β (1 ng/ml) and TNF-α (50 ng/ml) for 30 min with or without resveratrol (5, 25, and 125 μM). Glia were isolated from the brains of postnatal day 2 pups as described earlier. In brief, cerebral cortices were removed from the rat brains for glial cultures. The tissue was dissociated in 0.0025 % trypsin/EDTA and passed through a 70-mm pore nylon mesh. After centrifugation, the cell pellet was resuspended in DMEM containing 10 % FBS, 50 U/ml penicillin, and 50 mg/ml streptomycin. The cell culture dishes were coated with 10 μg/ml type I collagen (BD Biosciences, USA) for 12 h at 37 °C. Excess collagen was removed by PBS solution at 37 °C. Then, the glial cells in DMEM containing 10 % FBS were loaded into the dishes. After 7 to 10 days, glial cells typically reached 80 confluence and were ready for the experiment. In general, the cultures consisted of more than 95 % astrocytes (as indicated by GFAP-positive staining). The cells were activated with IL-1β (1 ng/ml) and TNF-α (50 ng/ml) for 24 h with resveratrol (5, 25, and 125 μM). As for the measurement of proteins in astrocytes, cells were collected 30 min after stimulation.

### Western blotting

To identify temporal expression of p-AMPK, GFAP, IBA-1, IL-1β, TNF-α, p-NR1, p-PKCγ, p-p38, p-ERK, p-JNK, and GAPDH, whole-cell protein extract lysates were used. Under anesthesia and immediately after perfusion with PBS, the rat spinal cord tissue was rapidly removed and homogenized. The filters were blocked with 5 % bovine serum albumin (BSA) and then incubated overnight at 4 °C with the primary antibodies (p-NR1 (Ser897), 1:800; p-AMPK (Thr172), 1:1000; TNF-α, 1:1000; p-ERK1/2 (Thr202/Tyr204), 1:1000; p-JNK (Thr183/Tyr185), 1:1000; p-p38 (Thr180/Tyr182), 1:1000; p-PKCγ (Thr514), 1:1000; GAPDH, 1:1000; GFAP, 1:500; IL-1β, 1:500; and IBA-1, 1:1000). The filters were developed using ECL reagents (PerkinElmer) with secondary antibodies from Millipore Bio-science Research Reagents. Data were analyzed with a Molecular Imager (Gel Doc TMXR, 170-8170) and the associated software Quantity One-4.6.5 (BioRad Laboratories).

### Immunohistochemistry

Under deep anesthesia, rats were transcardially perfused with PBS followed by 4 % paraformaldehyde with 1.5 % picric acid in 0.16 M PB (pH 7.2–7.4, 4 °C), and then, the rat spinal cord tissue was dissected out and postfixed in the same fixative overnight. The embedded blocks were sectioned (30 μm thick) and processed for immunofluorescence. Sections from each group (five rats in each group) were incubated with rabbit anti-c-Fos polyclonal antibody (1:100, sc-52, Santa Cruz Biotechnology), rabbit anti-calcitonin gene-related peptide (CGRP) polyclonal antibody (1:1000, Millipore), rabbit polyclonal anti-GFAP (1:500, ab7260, Abcam), and rabbit polyclonal anti-IBA (1:100, 019-19741, Wako Pure Chemical Industries), respectively. Rabbit IgG (1:200, Vector Laboratories) was used as an isotype control. For double immunofluorescence staining, the free-floating sections were incubated in PBS containing 10 % donkey serum and 1 % BSA for 2 h, incubated at 4 °C in primary antibody, then washed three times in 50 mM Tris-HCl (pH 7.4) PBS, and incubated in the secondary antibody either for 2 h at room temperature or overnight at 4 °C. After washing three times in PBS, sections were reincubated in blocking serum for 1 h. Morphologic details were examined with a confocal microscope (Leica TCS SP2). Images were randomly coded and transferred to a computer for further analysis. Fos-immunoreactive neurons were counted in a blind fashion. The number of Fos-like-immunoreactive neurons in the spinal trigeminal nucleus was determined by averaging the counts made in 20 spinal cord sections for each group. To obtain quantitative measurements of CGRP immunofluorescence, 15–20 fields covering the spinal trigeminal nucleus in each group were evaluated and photographed at the same exposure time to generate the raw data. Fluorescence intensities of the different groups were analyzed using MicroSuite image analysis software (Olympus America). The average green fluorescence intensity of each pixel was normalized to the background intensity in the same image.

### Statistical analyses

SPSS Rel 15 (SPSS Inc., Chicago, IL) was used to conduct all the statistical analyses. Alteration of the expression of the proteins was detected, the behavioral responses were tested with one-way ANOVA, and the differences in latency over time among groups were tested with two-way ANOVA. Bonferroni post hoc tests were conducted for all ANOVA models. Results are expressed as mean ± SEM of three independent experiments. Results described as significant are based on a criterion of *P* < 0.05.

## Results

### Resveratrol ameliorates mechanical allodynia in a trigeminal neuralgia rat model

**Fig. 1:**
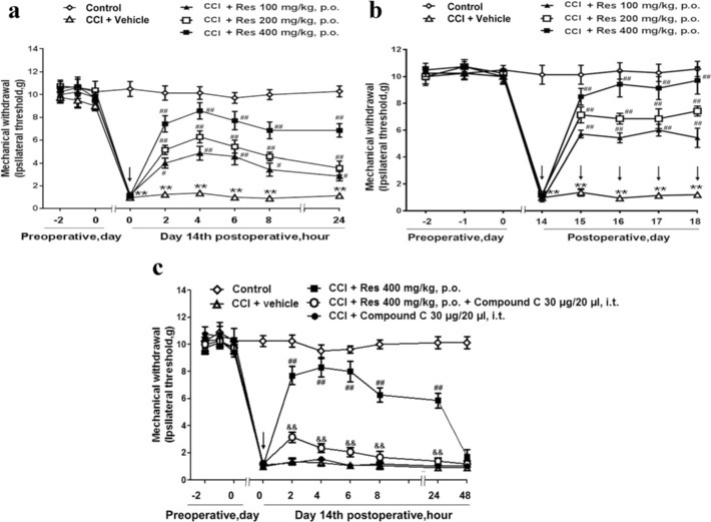
Resveratrol ameliorates mechanical allodynia in a trigeminal neuralgia rat model. Rats administered Res only on day 14 (**a**) or on days 14, 15, 16, 17, and 18 (**b**) post-CCI showed a significant increase in mechanical withdrawal compared with that of the control group. **a** Resveratrol (100, 200, and 400 μg/ml) orally single administrated at day 14 ameliorated pain-related behaviors in a dose-dependent manner, and this effect lasted for more than 24 h. **b** Consecutive administration of resveratrol at postoperative days 14, 15, 16, 17, and 18 reversed the mechanical allodynia in the CCI rats. **c** The AMPK inhibitor compound C reversed the effects of Res on CCI-induced mechanical allodynia. Drug administration is indicated by the *arrows* (*n* = 8 each group). Two-way ANOVA revealed a significant difference at *P < 0.05 and **P < 0.01 versus control; ^#^ *P* < 0.05 and ^##^ *P* < 0.01 versus the CCI group; and &*P* < 0.05 and &&*P* < 0.01 versus the CCI + Res 400 mg/kg group

In this study, rats that received CCI of the trigeminal nerve exhibited mechanical allodynia (Fig. 1). There were no significant differences in pain-related behaviors between the control group and the other groups before surgery. A marked decrease in mechanical withdrawal was observed 7 days after CCI and followed by a peak on day 14. Thus, resveratrol was administered to the CCI rats on day 14. These pain-related behaviors were greatly ameliorated by resveratrol (100, 200, and 400 μg/ml) orally single administrated at postoperative 14 days in a dose-dependent manner, and the effect lasted for more than 24 h (Fig. 1a). However, the effects of resveratrol were abolished by the AMPK inhibitor compound C (Fig. 1c). In addition, consecutive administration of resveratrol at postoperative days 14, 15, 16, 17, and 18 reversed the mechanical allodynia in the CCI rats (Fig. 1b). These results suggest that AMPK activation via resveratrol had a clearly positive effect on mechanic analgesia in the CCI rats.

### Resveratrol inhibits phosphorylation of c-Fos and CGRP in the spinal trigeminal nucleus

**Fig. 2:**
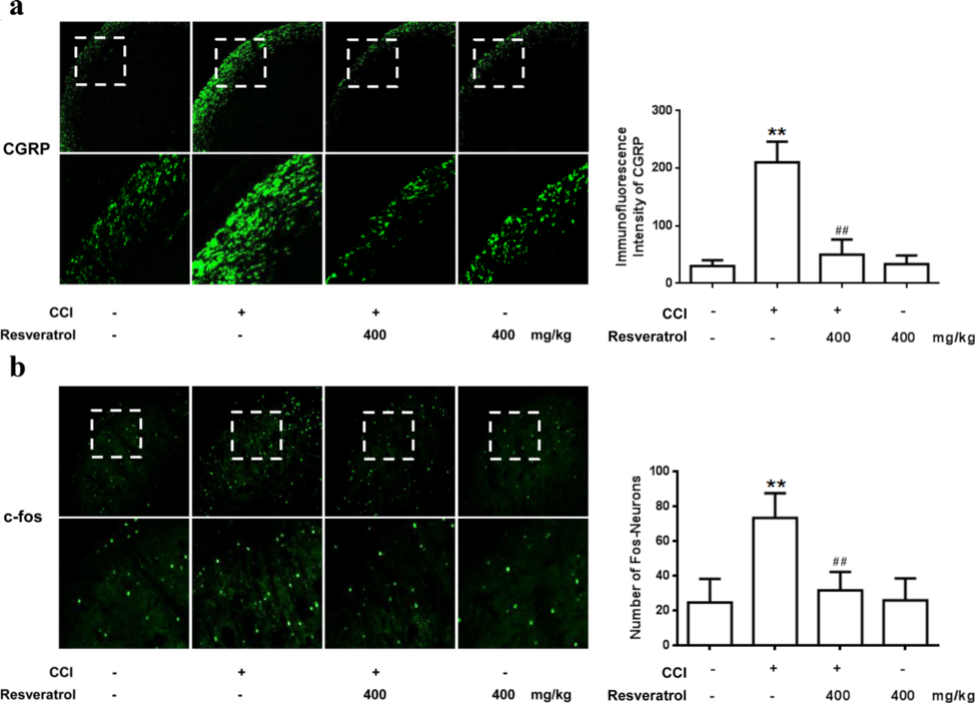
Resveratrol inhibits phosphorylation of c-Fos and CGRP in the STN. Immunofluorescence analysis data show CGRP expression and c-Fos-immunoreactive neuron number. The CGRP and c-Fos level was significantly increased by CCI, and resveratrol suppressed the increase of CGRP expression (**a**) and attenuated the increase of c-Fos-immunoreactive neuron number (**b**). Resveratrol alone had no effect on CGRP expression and c-Fos-immunoreactive neuron number. *n* = 5, five images per animal. *P < 0.05 and **P < 0.01 versus control; and ^#^ *P* < 0.05 and ^##^ *P* < 0.01 versus the CCI group

CGRP has an essential role in trigeminal nociceptive processing. As shown in Fig. 2a, significantly higher CGRP level was induced by CCI than that in the control group. Treatment with resveratrol suppressed the increase of the CGRP level. Resveratrol alone had no effect on the expression of CGRP. Furthermore, CCI remarkably increased the number of c-Fos-immunoreactive neurons in the spinal trigeminal nucleus (STN), and the increase of positive neurons was significantly attenuated by administration of resveratrol. Similarly, resveratrol alone did not influence the number of c-Fos-immunoreactive neurons in the STN in normal rats (Fig. 2b).

### Resveratrol inhibits the activation of microglia and astrocytes in rat STN

**Fig. 3:**
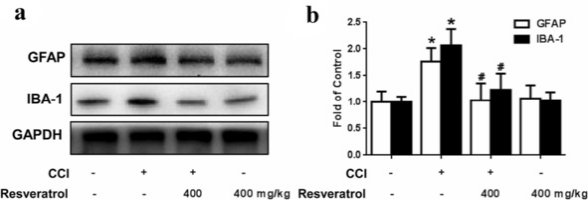
Resveratrol inhibits the activation of microglia and astrocytes in rat STN. **a**, **b** Representative western blot bands and a data summary (*n* = 4 each group) of the expression of GFAP and IBA-1, which are markers of astrocytes and microglia, respectively. GFAP and IBA-1 expression were enhanced in the STN of the CCI rats, whereas resveratrol (400 mg/kg, p.o.) downregulated the expression of both markers. *P < 0.05 and **P < 0.01 versus control; and ^#^ *P* < 0.05 and ^##^ *P* < 0.01 versus the CCI group

Astrocytes and microglia play a key role in the central sensitization process that occurs in neuropathic pain. The level of GFAP and IBA-1 was determined by western blotting and used as an indicator of astrocyte and microglia activation. To investigate whether resveratrol affects the activation of glia, we examined the levels of GFAP and IBA-1 in the STN. As shown in Fig. 3a and b, the expression of both GFAP and IBA-1 was enhanced in the STN of the CCI rats when compared with the control group, whereas resveratrol (400 mg/kg, p.o.) downregulated the expression of both markers (*P* < 0.05). These findings suggested that AMPK activation downregulated the activation of astrocytes and microglia in the CCI rat model.

**Fig. 4:**
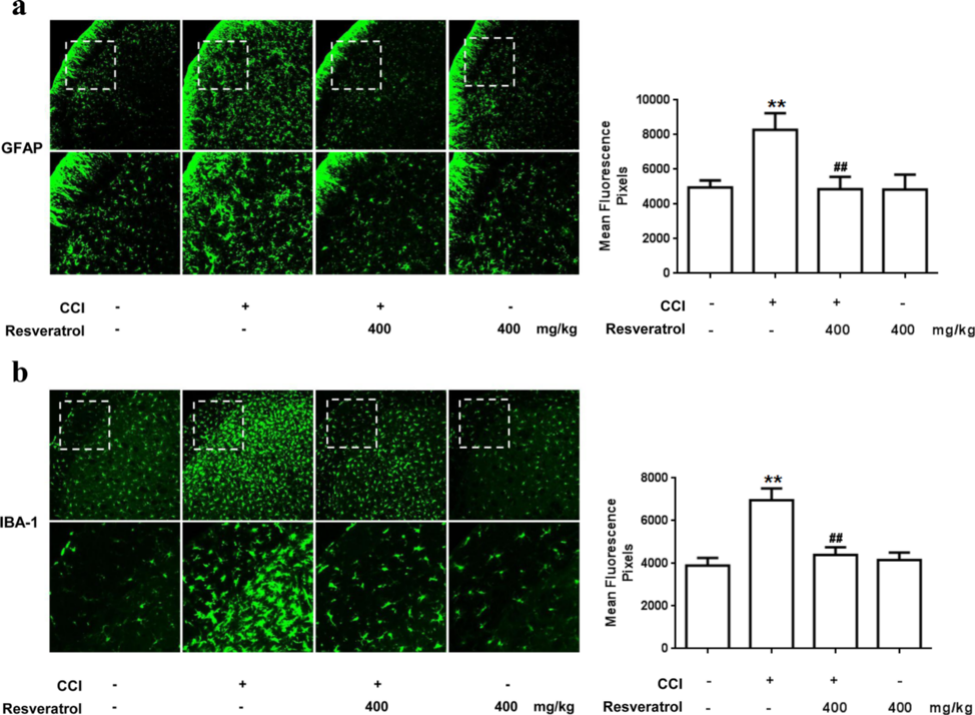
Resveratrol inhibits the activation of microglia and astrocytes in rat STN. **a**, **b** Immunofluorescence analysis data show GFAP and IBA-1 expression (*n* = 5, five images per animal). CCI increased GFAP and IBA-1 immunoreactivity in the STN, whereas resveratrol (400 mg/kg, p.o.) downregulated the expression of both markers. *P < 0.05 and **P < 0.01 versus control; and ^#^ *P* < 0.05 and ^##^ *P* < 0.01 versus the CCI group

In addition to immunoblotting, immunofluorescence staining was performed to confirm increased GFAP and IBA-1 immunoreactivity in the STN. As shown in Fig. 4a, b, compared with the control group, increased green fluorescence was observed in the STN ipsilateral to CCI, suggesting the activation of both astrocytes and microglia. Similar to the results from immunoblotting analysis, this activation was alleviated by resveratrol. These results supported the notion that AMPK was involved in regulating glial activation in TN.

### Resveratrol reduces CCI-induced production of pro-inflammatory factors and phosphorylation of NR1 and PKCγ in the STN

**Fig. 5:**
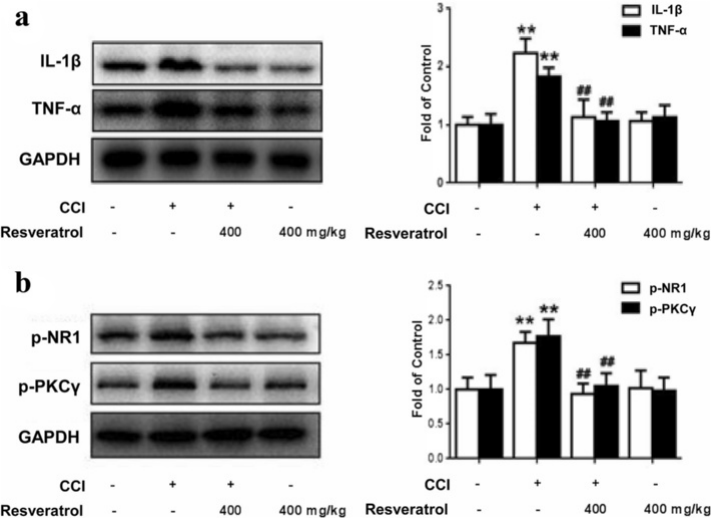
Resveratrol reduces CCI-induced production of pro-inflammatory factors and phosphorylation of NR1 and PKCγ in the STN. **a** Resveratrol reduces the production of pro-inflammatory factors IL-1β and TNF-α. **b** Resveratrol inhibits the phosphorylation of NR1 and PKCγ. Representative western blot bands and a data summary (*n* = 4 each group) are shown. *P < 0.05 and **P < 0.01 versus control; and ^#^ *P* < 0.05 and ^##^ *P* < 0.01 versus the CCI group

AMPK activation has been reported to inhibit neuroinflammation [21]. IL-1β and TNF-α are characterized pro-inflammatory cytokines that play an important role in inflammatory response by stimulating glial cells. The expression levels of IL-1β and TNF-α in the STN were examined by western blotting, showing that the levels of IL-1β and TNF-α were increased in the CCI rats compared to the control group (*P* < 0.01). Treatment with resveratrol (400 mg/kg, p.o.) inhibited the increased production of IL-1β and TNF-α in the STN (*P* < 0.01) (Fig. 5a). There were no significant differences between control and resveratrol alone.

The NMDA receptor, which regulates neuronal activity and synaptic efficacy, has a well-established role in various pain states. NMDA receptor phosphorylation can be activated by pro-inflammatory factors. The NMDA receptor 1 (NR1) subunit also can be phosphorylated by protein kinase C (PKC). Thus, we examined whether trigeminal nerve injury was able to elicit phosphorylated NR1 and PKCγ. Using CCI of a trigeminal nerve rat model, we found that CCI significantly increased the levels of phosphorylated NR1 and PKCγ, whereas repetitive treatment with resveratrol (400 mg/kg, p.o.) reversed the increase of their expression (Fig. 5b). Neither the production of pro-inflammatory factors nor the phosphorylation of NR1 and PKCγ was altered by resveratrol treatment alone in naïve rats.

### Resveratrol regulates phosphorylation of MAPK in vivo and in vitro via AMPK activation

**Fig. 6:**
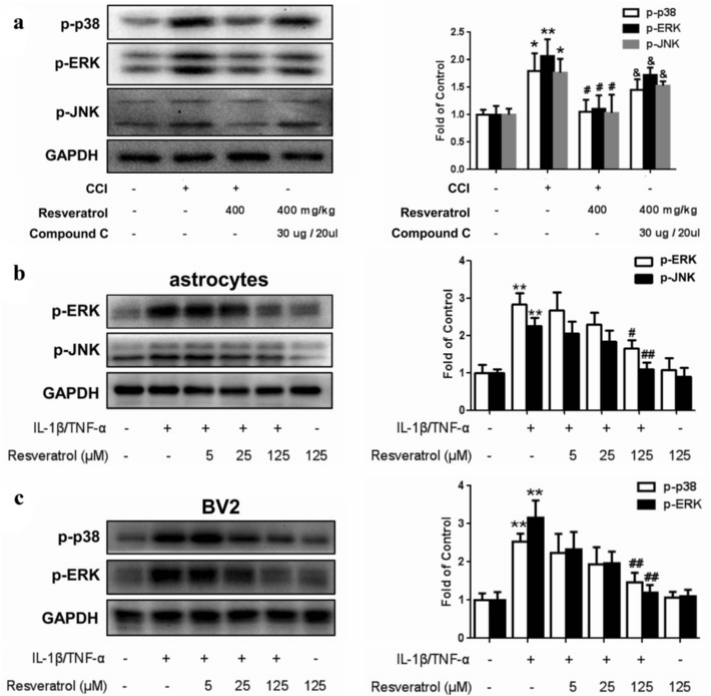
Resveratrol regulates phosphorylation of MAPK via AMPK activation in vivo and in vitro. **a** Resveratrol downregulates the phosphorylation of MAPKs in vivo, including p38, ERK, and JNK; these effects were reversed by compound C. Compound C (30 μg/20 μl, i.t.) was coadministered. **b** Resveratrol increased p-JNK and p-ERK expression in primary astrocytes in a dose-dependent manner. **c** Resveratrol increased p-p38 and p-ERK expression in BV2 cells in a dose-dependent manner. Western blot bands and a data summary (*n* = 4 each group) are shown. *P < 0.05 and **P < 0.01 versus control; and ^#^ *P* < 0.05 and ^##^ *P* < 0.01 versus the CCI group

Previous studies indicated that the production of both IL-1β and TNF-α might be mediated by MAPK signaling pathways in glia [22, 23]. As shown in Fig. 6a, administration of resveratrol (400 mg/kg, p.o.) significantly reduced the levels of phosphorylated MAPKs in the STN, including p38, extracellular signal-regulated protein kinase (ERK), and c-Jun N-terminal kinase (JNK), induced by CCI. These effects were reversed by the co-administration of the AMPK inhibitor compound C (30 μg, i.t.). These results suggested that resveratrol-mediated suppression of MAPKs may be secondary to AMPK signaling activation.

Our findings suggested that resveratrol may act through MAPK signaling pathways to attenuate CCI via the inhibition of spinal cord glial activation in vivo. We further sought to expose cultured primary astrocytes from newborn rats to IL-1β (1 ng/ml)/TNF-α (50 ng/ml) to mimic the effect of neuroinflammation in rats to investigate the role of resveratrol. IL-1β/TNF-α treatment induced activation in astrocytes and was characterized by increased phosphorylation of JNK and ERK/MAPKs. Treatment with resveratrol (5, 25, and 125 μM) before IL-1β/TNF-α administration significantly reduced these effects (Fig. 6b).

To investigate the in vitro effects of resveratrol on pro-inflammatory factor-induced microglia activation, we used the immortalized murine microglial cell line BV-2 to examine the effect of resveratrol on primary mouse microglia cells [24, 25]. BV-2 cells have a similar reaction pattern to that of primary microglia after stimulation with lipopolysaccharide [26] and have been used in vitro to replace primary microglia, which pose experimental difficulties when being separated from the STN in vitro. In the present study, IL-1β (1 ng/ml)/TNF-α (50 ng/ml) treatment induced BV-2 cell activation, which was characterized by increased phosphorylation of p38 and ERK/MAPKs (Fig. 6c). Treatment with resveratrol (5, 25, and 125 μM) before IL-1β/TNF-α administration significantly reduced these effects.

## Discussion

Our results provide several novel insights into the pathology and potential treatment of TN. We have demonstrated that resveratrol suppresses mechanical allodynia of the trigeminal nerve and increases the expression of CGRP and c-Fos induced by CCI in rats. Resveratrol had a significant impact on the CCI-induced activation of astrocytes and microglia and decreased the expression level of pro-inflammatory cytokine in TN. Resveratrol reduced the upregulated phosphorylation of MAPKs both in vivo and in vitro in an AMPK-dependent manner. These studies provide an assessment of resveratrol for TN and for the future development of more efficacious AMPK activators for the treatment of chronic trigeminal pain.

Infraorbital nerve (the second branch of the trigeminal nerve) ligation is a validated model for producing allodynia in the ipsilateral vibrissae area in rats [27]. In the present study, we have revealed that resveratrol, an AMPK activator, produced a marked increase in pressure threshold to evoke a nocifensive response in trigeminal nerve-ligated rats (Fig. 1). Furthermore, we noted that CCI of the trigeminal nerve induced upregulation of CGRP in the STN (Fig. 2). The neurotransmitter CGRP plays a crucial role in the pathophysiology of neuropathic pain-related behavior in the CCI rats [27]. It is well known that activation of the trigeminal nerve system induces the release of CGRP [28]. Our group has demonstrated that the increase of CGRP release induced by CCI of the trigeminal nerve could be almost completely abolished by reducing the central sensitization of primary trigeminal sensory neurons with resveratrol treatment. We observed a similar pattern in the CCI of the trigeminal nerve, with the number of the immediate early gene c-Fos IR cells increased remarkably, indicating the activation of the trigeminal system. Our findings correspond well with a previous report that c-Fos in the STN was elevated after stimulation of the infraorbital nerve [29]. Treatment with resveratrol was shown to effectively block c-Fos upregulation. Previous data demonstrated that resveratrol significantly alleviated the behavior and pathophysiology of trigeminal neuropathic pain. However, the mechanism by which resveratrol exerts an analgesic effect in TN has not been illuminated.

Trigeminal neuropathic pain following trigeminal nerve injury is often difficult to diagnose and treat due to complexity of TN mechanisms [30–33]. Accumulating evidence suggests that translation regulation at the level of the primary afferent neuron is vital for the establishment and maintenance of enhanced pain states [34, 35]. Neuropathic pain treatment is currently aimed only at reducing symptoms, generally by suppressing neuronal activity. In contrast, targeting neuroglia may provide more opportunities for disease management by aborting neurobiological alterations that support the development of persistent pain. Multiple lines of evidence show that glia cells, notably microglia and astrocytes, contribute to the central sensitization process that occurs in the peripheral nerve injury. The activated microglia and astrocytes release numerous substances, including pro-inflammatory cytokines such as IL-1β, TNF-α, and COX-2 [36–38]. IL-1β is one of the most vital cytokines, and directly sensitizes the nociceptors, such as the transient receptor potential cation channel subfamily V member 1 (TRPV1), a heat and chemical-sensitive cation channel, in primary sensory neurons [39]. It has been reported that the upregulation of TNF-α in the DRG and spinal dorsal horn is induced by injury of the periphery nerve [40–42]. It is possible that TNF-α also participates in neuropathic pain. Our study demonstrated that peripheral injury of the trigeminal nerve also activated glial activation and enhanced the secretion of IL-1β and TNF-α (Figs. 3 and 4a). The influence of resveratrol on other pro-inflammatory cytokines in TN will be the focus in our future work.

AMPK has been regarded as the energy sensor for more than one decade [43, 44]. AMPK is a ubiquitous kinase endogenously activated by AMP and ADP and exogenously regulated by a variety of pharmacological entities including the widely prescribed anti-diabetes drug metformin and natural products such as resveratrol [45]. AMPK is regarded as a regulator of neuronal function, plasticity, and neurodegeneration [46, 47], but the potential mechanisms of AMPK activation on neuronal excitability, as an important component of neuropathic pain conditions [48], is not known completely. Previous studies mostly focus on discovering the mechanism of effect of activating AMPK on sensory neuronal excitability, with few reports on the regulation of AMPK activation on the function of glia in neuropathic pain. It was reported that AMPK activation in the spinal glia by resveratrol participates in the treatment of tumor cell implantation-induced neuroinflammation [49]. Moreover, pharmacological activation of AMPK enhanced glial glutamate transporter activity by attenuating glial glutamate transporter-1 internalization in neuropathic pain mice [50]. Our study revealed that resveratrol could activate AMPK to inhibit CCI-evoked astrocyte and microglial activation and reversed the production of IL-1β and TNF-α (Figs. 3 and 4a). These results have well documented the role of AMPK in neuroglial cells. We validated the hypothesis that AMPK activation could inhibit glia activation and relieve glia-mediated neuroinflammation, and thus alleviate trigeminal neuralgia (Figs. 1, 2, 3, and 4a), which is in agreement with previous related studies [51, 52].

The NMDA receptor has an ionotropic property which regulates Ca^2+^ influx and Ca^2+^-dependent physiological effects, further neuronal activity, and synaptic efficacy. Resveratrol administration reversed the production of pro-inflammatory cytokines IL-1β and TNF-α which regulate synaptic plasticity. It is widely accepted that the phosphorylation of NMDA receptors (NMDARs) also has a momentous role in neural plasticity and various pain states [53]. The activation of PKCγ plays a well-developed role in central sensitization in neuropathic pain, which may contribute to increasing the excitability of nociceptive neurons [54]. Our results showed that resveratrol inhibited in the phosphorylation of NMDAR NR1 and the activation of PKCγ in the STN in the CCI rats (Fig. 4b). Thus, we provided the evidence for the first time that resveratrol administration in the peripheral nerve may inhibit glial activation and relieve glia-mediated neuroinflammation via AMPK activation, and further suppress central sensitization present in the STN, which may explain why resveratrol has a sustaining analgesic effect.

MAPK plays a key role in the induction and maintenance of neuropathic pain [55]. MAPK signal transduction pathways are evolutionarily conserved in eukaryotic cells and transducer signals in response to a variety of extracellular stimuli [56]. Among the well-characterized MAPK subfamilies, the p38 kinases are first defined for drugs inhibiting TNF-α-mediated inflammatory response [57]. Because the MAPKs are vital regulators of the expression of many cytokines, they appear to be involved in neuroinflammation of TN. Our studies demonstrated that the significantly increased phosphorylative level of MAPKs in the STN was induced by CCI. Oral administration of resveratrol downregulated the expression levels of MAPKs (Fig. 5a). We also verified the change trend of the phosphorylation of MAPKs in primary astrocytes and microglia cell line BV-2 cell and obtained consistent results (Fig. 5b, c). These results indicated that MAPKs in astrocytes and microglia might be involved in the mechanism of resveratrol managing the glial activation and release of inflammatory cytokines. Our results were consistent with other relevant reports that resveratrol affords anti-neuroinflammatory effects by inhibiting microglial activation after suppressing the activation of MAPK signaling pathways [33, 40, 58].

## Conclusions

In this study, we have found a novel pathway for the potential treatment of TN, activating glial AMPK. Pharmacological AMPK activation resolves trigeminal neuropathic allodynia and decreases sensory neuron excitability by inhibiting glial activation and release of cytokines, which is regulated by MAPK pathway. Due to natural production and safety of resveratrol, these preclinical results have the potential to be rapidly translated into the clinic.
